# Overestimation of Vitamin A Supplementation Coverage from District Tally Sheets Demonstrates Importance of Population-Based Surveys for Program Improvement: Lessons from Tanzania

**DOI:** 10.1371/journal.pone.0058629

**Published:** 2013-03-11

**Authors:** Christina Nyhus Dhillon, Hamsa Subramaniam, Generose Mulokozi, Zo Rambeloson, Rolf Klemm

**Affiliations:** 1 Helen Keller International-Tanzania, Dar es Salam, Tanzania; 2 Center for Human Nutrition, Department of International Health, Bloomberg School of Public Health, Johns Hopkins University, Baltimore, Maryland, United States of America; 3 A2Z: The USAID Micronutrient and Child Blindness Project, AED (FHI360), Washington, DC, United States of America; 4 Tanzania Food and Nutrition Centre, Dar es Salam, Tanzania; Aga Khan University, Pakistan

## Abstract

**Background:**

Tanzania has conducted a national twice-yearly Vitamin A supplementation (VAS) campaign since 2001. Administrative coverage rates based on tally sheets consistently report >90% coverage; however the accuracy of these rates are uncertain due to potential errors in tally sheets and their aggregation, incomplete or inaccurate reporting from distribution sites, and underestimating the target population.

**Objectives:**

The post event coverage survey in Mainland Tanzania sought to validate tally-sheet based national coverage estimates of VAS and deworming for the June 2010 mass distribution round, and to characterize children missed by the national campaign.

**Methods:**

WHO/EPI randomized cross-sectional cluster sampling methodology was adapted for this study, using 30 clusters by 40 individuals (n = 1200), in addition to key informant interviews. Households with children 6–59 months of age were included in the study (12–59 months for deworming analysis). Chi-squared tests and logistic regression analysis were used to test differences between children reached and not reached by VAS. Data was collected within six weeks of the June 2010 round.

**Results:**

A total of 1203 children, 58 health workers, 30 village leaders and 45 community health workers were sampled. Preschool VAS coverage was 65% (95% CI: 62.7–68.1), approximately 30% lower than tally-sheet coverage estimates. Factors associated with not receiving VAS were urban residence [OR = 3.31; p = 0.01], caretakers who did not hear about the campaign [OR = 48.7; p<0.001], and Muslim households [OR<3.25; p<0.01]. There were no significant differences in VAS coverage by child sex or age, or maternal age or education.

**Conclusion:**

Coverage estimation for vitamin A supplementation programs is one of most powerful indicators of program success. National VAS coverage based on a tally-sheet system overestimated VAS coverage by ∼30%. There is a need for representative population-based coverage surveys to complement and validate tally-sheet estimates.

## Introduction

An estimated 42% of children below five years of age in sub-Saharan Africa are at risk of VAD.[1] Vitamin A supplementation is widely accepted as a critical intervention to reduce child mortality.[2] Achieving consistent coverage over 80% is necessary to achieve the mortality reduction demonstrated by efficacy studies. [3] Some countries have achieved high coverage of twice-yearly distribution of vitamin A capsules to preschool aged children (6–59 months old) children; however other countries struggle to reach and sustain high VAS coverage. [4] VAS coverage estimates provide a reasonable measure of assessing program success considering the cost, invasiveness and logistical difficulties of blood collection, storage and analysis, the difficulties of interpreting biological markers of VA, and the poor responsiveness of serum retinol to VAS.

Coverage estimates can come from tally sheets completed during the distribution, with the data incorporated into the health information system (HMIS) or from district or national level representative population-based surveys, or possibly sentinel surveillance.

Most countries use a tally sheet system, which records the doses administered at a health post or other location, and then aggregates these upward to the next level (e.g. district, region, nation) to provide a total numerator (i.e. total number of capsules distributed). When applied to the total number of children in the target age group at each level, this provides a coverage estimate for any given round. This approach can provide valuable information allowing coverage comparisons by district, region or country over time, and be used to identify and focus attention on low coverage areas. Tally systems, however, can also suffer from some intrinsic weaknesses related to inaccurate population estimates for a given location, human error in counting and calculating, and delayed, incomplete or missing reports from districts leading to inaccurate coverage estimates.

For national coverage estimates, it has been common in recent years to have some questions on receipt of VAS included in large representative population-based national surveys such as DHS or UNICEF's Multiple Indicator Cluster Surveys (MICS). These surveys provide ‘benchmark’ national and regional coverage estimates, depending on the sampling frame, but often do not provide district coverage estimates. Cost of mounting such surveys prohibits semi- or annual estimates, and coverage estimates can be affected by the timing of the survey in relation to VAS and survey questions and methods that allow respondents to differentiate VAS from other interventions.

Since 2001, mainland Tanzania has conducted twice-yearly national-scale VAS campaigns as a response to its documented high prevalence of VAD and under- five mortality. Tanzania has used a tally sheet system to estimate coverage and has consistently reported VAS coverage greater than 80 percent. Previous representative population-based surveys by Helen Keller International in 2004 and 2006 confirm high but slightly overestimated (4–8%) coverage estimates than those reported by the Tanzania tally system.[5] Another VAS post-event coverage survey was conducted immediately after the June 2010 campaign aimed to estimate VAS coverage in Mainland Tanzania among children 6–59 months and de-worming coverage among children 12–59 months. This survey also provided the opportunity to assess factors associated with children receiving or not receiving a vitamin A supplement, and to examine barriers to VAS participation. In this paper we report on these findings for VAS coverage estimates and risk factors associated with not receiving a vitamin A supplement.

## Methods

### Ethics Statement

Ethical approval for conducting the survey was provided by the Tanzanian National Institute of Medical Research (NIMR) and the Institutional Review Board (IRB) of the Academy for Educational Development (now part of FHI360) in Washington, DC. Consent was first obtained from village or urban neighborhood leaders by survey supervisors. Subsequently, individual verbal informed consent was obtained from the caretaker in each surveyed household in accordance to local standard practice for participation in observational studies. Enumerators recorded the outcome of the consent procedure on the survey form on behalf of the participant. This procedure was approved by NIMR and the IRB of the Academy for Educational Development.

### Study Design

This study was a nationally representative population-based cross-sectional cluster survey among households with children 6–59 months of age in mainland Tanzania.

### Sampling

Thirty clusters were randomly selected using probability proportional to size (PPS). The primary sampling unit was a registered village (cluster)- the smallest unit for which there is population data from the Tanzania National Bureau of Statistics (NBS) data. Clusters were selected by systematic randomization using an interval obtained by dividing the cumulative population by the desired 30 clusters, with the first village in the interval selected by a random start.

### Data Collection

Six teams of six enumerators with a team leader and supervisor for every two teams were employed to conduct the survey. After orienting district health offices and village leaders about the survey, each survey cluster was divided into 4 quadrants using a local map. In each quadrant, one of 5 starting points was chosen at random. At each starting point, a bottle was spun to determine the direction of the households for selection. Once the direction was determined, the number of households from the starting point to the end of the quadrant in the direction of the bottle was estimated and a house was selected at random as the starting household. Following the direction of the bottle spin from this first household, the next 10 eligible households were interviewed. This process was repeated in each of the 4 quadrants of the cluster to secure a total of 40 households with a child 6–59 months of age per cluster.

Households were screened for eligibility based on having a preschool child at the time of the June 2010 round of supplementation. Within each eligible household, only one eligible child was selected to be the focus of the survey. If multiple children of eligible age lived in the household, one child was chosen at random. Child ages were verified by health cards whenever possible and when unknown, were estimated using life event calendars. This method was repeated in all quadrants in each of the 30 clusters sampled across mainland Tanzania resulting in a total estimated sample size of 1,200 households. In order to assist informants to recall vitamin A supplements, interviewers showed samples of capsules of different doses normally used during distribution rounds during visits.

After obtaining consent, the care taker of the selected child was interviewed about measles vaccination; uptake of vitamin A and deworming in the June 2012 campaign; uptake of vitamin A in rounds other than the June 2012 round; barriers to VAS; post-partum VAS; infant and young child feeding practices; utilization of health care services; knowledge about VAS benefits; and demographic information. Enumerators displayed vitamin A capsules when asking mothers about vitamin A and deworming to avoid possible confusion with other health interventions. The rapid assessment of the VAS coverage was completed within 6 weeks of the June/July 2010 round of distribution, increasing the accuracy of responses since the period of recall is relatively short. As some districts distributed VAS late in July, data collection was completed in July and August 2010.

In addition to caretakers/children in the sample, three other types of informants were sampled in each cluster and included 1–2 health workers (HW), 1–2 VAS community health workers (CHW) and 1 village/community leader. These interviews were conducted by team leaders and began with the village/community leader survey in each site. After this initial interview, two health workers per cluster were interviewed to collect additional data on the recent round of supplementation and assess general health worker characteristics and knowledge of VAS. As with the selection of children, names of potential informants were randomly selected. The health workers interviewed were randomly selected across the facilities within the cluster but had to be involved with VAS distribution in some way. The same random selection methodology was used to select two CHWs per cluster to interview using the names of all CHWs engaged in VAS provided by the village leaders. Most interviews took between 30–45 minutes. Requisite ethical approvals for conducting the survey were obtained prior to data collection.

A total sample size of 1,200 households was estimated to provide a national coverage estimate within ±5% of true coverage.

### Statistical Analysis

Analysis was restricted to children aged 6–59 months of age at the time of the 2010 distribution round in the district, for whom the status of VAC receipt during the last round was known. Districts did not distribute VAS on the same date therefore duration of distribution could vary up to one month. Furthermore, VAS receipt was not always recorded on the child health card. Therefore, age at distribution was calculated using a common distribution date of June 16, 2010 with a one-month leeway period.

All data were double entered into an EPI Info software system.[6] Data were then compared for errors and corrected by reviewing the original data form. Analysis was done using STATA statistical software.[7] Standard error calculation of coverage rates for VAS and de-worming were adjusted for survey design methodology using the STATA *svy* procedure. Chi-squared tests and simple logistic regression analysis were run to test differences among missed and covered children on various characteristics, including socioeconomic status (SES), maternal age and education, child age and sex, distance from health center, and religion.

Since expenditures were not estimated in the survey, SES of the household was determined using a wealth index.[8] The wealth index calculation included data on a household's ownership of assets and on available water and sanitation. Each asset and service variable was broken down into a dichotomous or categorical variable depending on whether or not the household owned that asset or used that service [8]. The variables were then processed in order to obtain their scoring factor or weight. The resulting population was then divided into wealth quartiles representing proxies for SES (i.e., lowest, lowest-middle, upper-middle, and upper). Wealth quartiles are thus expressed in terms of quartiles of households of the total population at risk for all measures. A p-value of <0.05 was considered significant.

## Results

**Figure 1:**
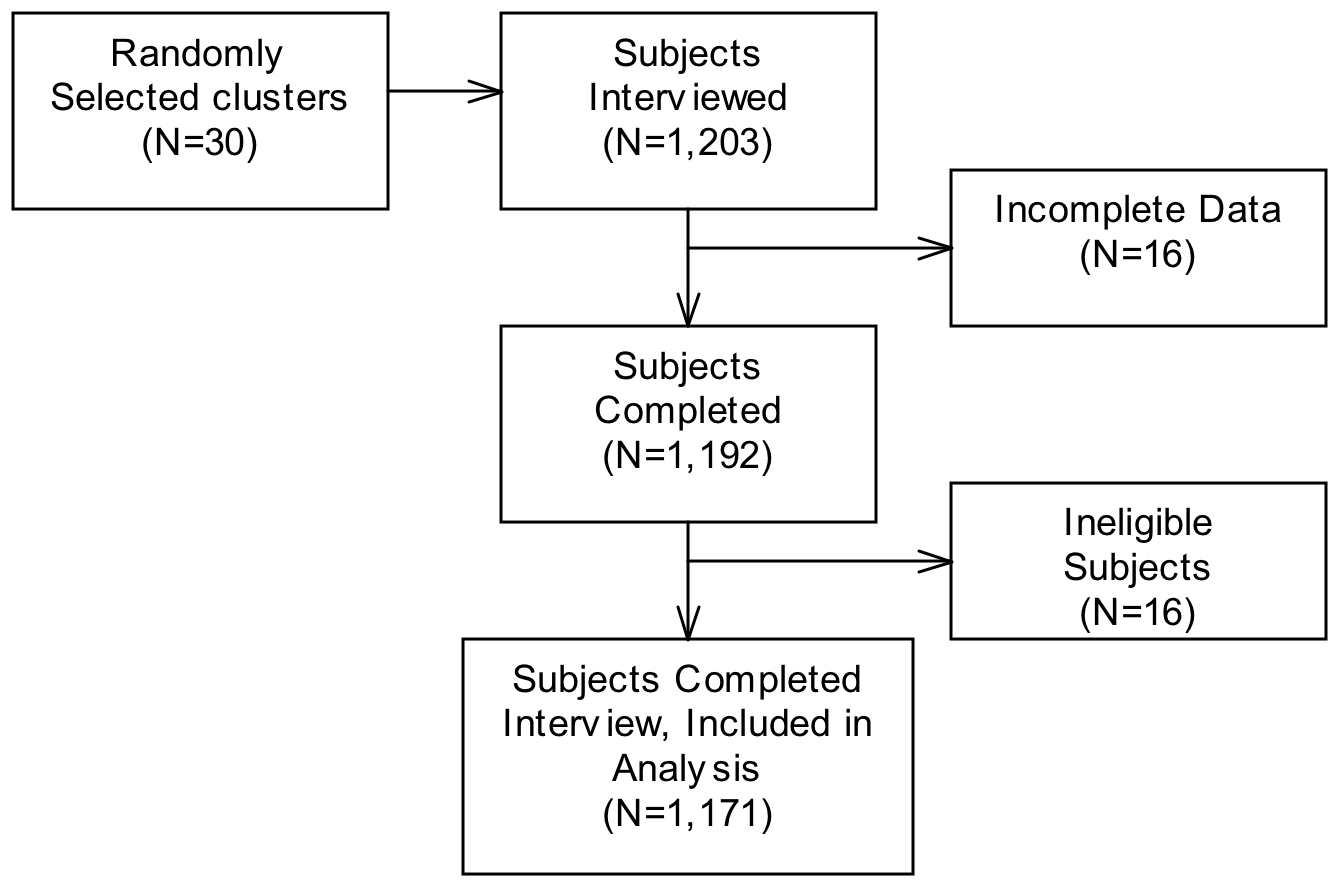
Flow of participants in final analytical sample.

A total of 1,203 households with children 6–59 months of age were interviewed, of which 1,171 (97.3%) were included in the final analytic sample (Figure 1). In addition, 58 government health workers, 30 village leaders, and 45 community health workers were interviewed.

The sample of children was equally distributed across 6-month age categories and gender, with slightly higher representation among younger children. Although all under-five children were to be included in the random selection, older children who were out of the home at the time of the survey may have been missed by the mother when asking for child names and ages. Ninety-nine percent of informants/caretakers were mothers.

### Overall VAS and Deworming Coverage

VAS coverage among children 6–59 months of age during the June 2010 round was 65% (95% CI: 62.7–68.1) as shown in Table 1 - approximately 30% lower than administrative coverage data, showing 98.5% coverage. Table 1 also shows that there are many eligible children who had never received VAS during any previous round according to the health card and/or caretaker recall (9.3%, CI: 8.1–11.4). There was no statistical difference in coverage by child sex or child age for VAS receipt.

**Table 1:** Child, Maternal and Household Characteristics, Children 6–59 months.

Characteristics		% Receive Vitamin A		% Did not Receive Vitamin A		Odds Ratio	95% CI	*p-value*
		(n/N) %		(n/N) %				
Overall VAS Coverage, June 2010		766/1171	65.41	405/1171	34.6		62.69, 68.14	
Overall VAS Coverage, Lifetime		1059/1168	90.7	109/1168	9.3		88.99, 92.34	
**Child Characteristics**								
Mean Age in Months [SD]: 28.97 [15.13]								
Age in months								
	6–11	110/166	66.3	56/166	33.7	1		
	12–23	212/310	68.4	98/310	31.6	1.10	(0.73, 1.65)	0.63
	24–35	193/272	71.0	79/272	29.0	1.24	(0.74, 2.10)	0.40
	36–47	132/226	58.4	94/226	51.6	0.71	(0.46, 1.12)	0.14
	48–60	118/195	60.5	77/195	39.5	0.78	(0.46, 1.33)	0.35
Sex								
	Male	363/569	63.8	206/569	36.2	1		
	Female	398/595	66.9	197/398	33.1	1.15	(0.89, 1.47)	0.27
**Maternal Characteristics**								
Mean Age in Years [SD]: 29.43 [7.79]								
Education								
	None	146/212	68.9	66/212	31.1	1		
	Primary Education	538/836	64.4	298/836	35.7	0.82	(0.49,1.37)	0.43
	Secondary and Above	33/50	66.0	17/50	34.0	0.88	(0.40, 1.92)	0.74
Number of Living Children								
	1	119/198	60.1	79/198	39.9	1		
	2	147/250	58.8	103/250	41.2	0.95	(0.67, 1.31)	0.73
	3	147/223	65.9	76/223	34.1	1.28	(0.83, 1.99)	0.25
	4	101/149	67.8	48/149	32.1	1.40	(0.87, 2.24)	0.16
	≥5	203/278	73.0	75/278	27.0	1.80	(1.17, 2.76)	0.01**
Received VAS Information Before Round								
	Yes	537/630	85.2	93/630	14.7	1		
	No	21/207	10.1	186/207	89.9	51.14	(26.10, 100.21)	<0.001**
**Household Characteristics**								
Urban/Rural								
	Urban	117/268	43.7	151/268	56.3	1		
	Rural	649/903	71.9	254/903	28.1	3.30	(1.55, 7.03)	0.003**
Religion								
	Muslim	139/315	44.1	176/315	55.9	1		
	Roman Catholic	273/368	74.2	95/368	25.8	3.63	(1.66, 7.97)	0.002**
	Non-Catholic Christian	308/428	72.0	120/428	28.0	3.25	(1.44, 7.34)	0.006**
	Traditional	40/51	78.4	11/51	21.6	4.60	(1.85, 11.47)	0.002**
	Other	6/9	66.7	3/9	33.3	2.53	(0.43, 14.97)	0.294
Income Quartile								
	First (Lowest)	94/136	69.1	42/136	30.9	1		
	Second	260/401	64.8	141/401	35.2	0.82	(0.51, 1.33)	0.42
	Third	268/370	72.4	102/370	27.6	1.17	(0.62, 2.21)	0.61
	Fourth (Highest)	144/264	54.5	120/264	45.5	0.54	(0.23, 1.24)	0.14
Main Source of Income								
	Farming	560/822	68.1	262/822	31.9	1		
	Business	114/188	60.6	74/188	39.4	0.72	(0.41, 1.24)	0.23
	Formal Employment	42/68	61.8	26/68	38.2	0.76	(0.33, 1.73)	0.50
	Informal Employment	15/38	39.5	23/38	60.5	0.31	(0.11, 0.84)	0.02*
	Other	35/54	64.8	19/54	35.2	0.86	(0.32, 2.25)	0.75

* statistical significance at p≤0.05; **statistical significance at p≤0.01

Mebendazole deworming tablet is offered to children 12–59 months of age alongside VAS during the twice-yearly supplementation. 96% of children receiving VAC also received mebendazole. Assuming children who did not receive VAS also did not receive mebendazole, deworming coverage of eligible children (12–59 months of age) was only 59% (CI: 54.0–64.1). The current policy of mebendazole administration for twice-yearly prophylactic deworming is specific to children 12–59 months of age. However data indicates that 24% of children were de-wormed against the current policy, having been supplemented when they were younger than 12 months.

### Characteristics of Missed Children

Over half (53%) of the children who missed the campaign did so because their caretaker was unaware of the campaign. Other commonly cited reasons for missing the campaign included not having a caretaker available to take the child (13%) and child not available during campaign (12%). Lack of supplies at the health facility and the journey being too far were cited by less than 5% of informants/caretakers.

Among caretakers of missed children, the distance to services or cost of reaching services were not significant reasons for non-participation. However, data indicated that average travel time to services were significantly different between missed and reached children. The average time to reach a VAS post was 26 minutes among children reached and 41 minutes among children missed in the last round (p<0.001). Twenty-five percent of the 81 ‘other’ reasons for missing the campaign indicated that the caretaker did not take the child for VAS because the child had not yet reached nine-months of age. Additional reasons for missing campaign included: child was ill; refusal, or don't care/not important.

Of the children who were missed by the June 2010 campaign (n = 413), 99% attended the clinic for other reasons. Therefore most of these children were receiving some type of health services. Reasons for attending the clinic among children missed by VAS included routine checkups (80%), vaccinations (28%) and sick visits (51%). Table 1 shows that a child with 4 or more siblings was approximately 40% more likely to receive VAS than a child with no siblings (OR = 1.62; p = 0.02).

A child from a non-Muslim household was over 3 times more likely to be supplemented than a child from a Muslim household (range of ORs =  3.25–3.91; p<0.01). A child living in a rural area was over 3 times more likely to receive VAS than a child living in an urban area (OR = 3.31; p = 0.01). Reduced coverage in urban areas, including Dar es Salaam, have been consistent for many rounds. There was no difference in coverage between missed versus reached children across age groups, child gender, maternal age and education, and household income quartile.

Approximately 10% of eligible children had never been supplemented by any previous round. The mean age of these ‘chronically missed’ or hard-to-reach children was 24 months (95% CI: 21.1–27.4). Reasons for being missed were similar between chronically missed children and children missed by the June 2012 round. However, a higher percentage of mothers cited ‘no one available to take child to campaign’ (18%) and travel time to the nearest health facility was significantly greater (49.5 minutes versus 29.4 minutes; p<0.01) in the chronically missed children.

### Caretaker Knowledge of VAS

Among caretakers interviewed, 44% knew that vitamin A could help protect a child from disease, and only half (49%) knew the benefits of vitamin A. Similarly, food sources of vitamin A were mostly unknown (45%), although one third of mothers knew that green leafy vegetables were a source of vitamin A. More caretakers heard about the VAS campaign through their community leaders (35%) than through community health workers (21%or health workers themselves (20%). About 15% of caretakers heard about the VAS campaign through loudspeaker announcements on roaming vehicles.

### Health Worker, Community Health Worker and Village Leader Knowledge of VAS

**Figure 2:**
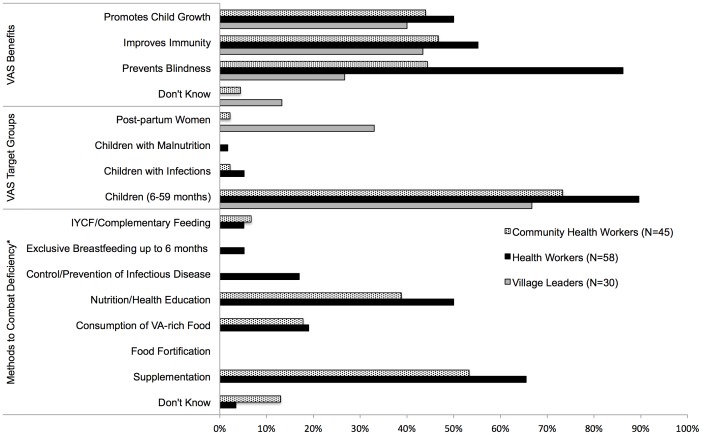
Knowledge of Vitamin A Among Village Leaders, Healthcare Workers and Community Health Workers. *Village leaders were not asked questions pertaining to methods of combatting VAD

Sixty-three percent of health workers (HWs) were females and represented all levels of healthcare providers. On average, among the surveyed HWs, the average years of service was 11.1 years (95% CI: 8.3–13.9) and 85% were working in a government health facility. (Figure 2)

The primary source of information about VAS for health workers was predominantly from their formal professional training (78%) with some mentioning workshops (22%) and job aids (12%). No health worker reported having received his or her VAS information from the national VAS implementation guidelines.

Only 32% of health workers had received any training in vitamin A supplementation, and 37% knew that Vitamin A supplements should not be given during pregnancy. Almost 90% of HWs knew the correct target for twice yearly VAS campaigns were 6–59 month old children. However, there was little knowledge that VAS should be provided to children with infections, children presenting with malnutrition. No informants in either group cited food fortification as a way to prevent VAD, and CHWs were unaware that breastfeeding is the main source of vitamin A for children below six months of age. Overall, nutrition knowledge among health workers and community health workers was poor.

## Discussion

A discrepancy of 30 percentage points was found between these preschool VAS coverage findings and the 98.5% reported by tally sheet data. The Demographic and Health Surveys (DHS) sponsored by USAID's MEASURE project found that average coverage over the June and December 2010 rounds were 59.8% [9], confirming the coverage survey findings. These recent government-reported data are highly overestimating VAS coverage.

The gross discrepancy in coverage rates may be to due to a number of factors including health worker measurement errors in summarization of the tally sheets, and underestimated target population figures, which are based on 2002 census projections. The 2012 census in Tanzania will help reduce improve the accuracy of tally sheet data in the future. Coverage estimation for vitamin A supplementation programs is one of most powerful indicators of program success. Due to the prevailing conditions, there remains the need for repeated representative population-based coverage surveys to complement and validate tally-sheet estimates to track program progress.

Understanding why and which children are missing this basic health service is also critical for program improvement. It is of great concern that 1 in 10 mainland Tanzanian pre-school age children have never been reached by VAS in their lifetime, despite the regular occurrence of twice-yearly events in Tanzania since 2001. Identification and provision of services to these hard-to-reach children is critical as they likely suffer disproportionately higher rates of mortality compared to other children.

The majority of missed children had caretakers who were unaware of the distribution event; therefore, it is important that timely social mobilization efforts provide caretakers with information about VAS and deworming. In addition, special emphasis should be placed on sensitizing Muslim populations on the importance of Vitamin A for reducing mortality and morbidity in preschool aged children. Unfortunately, funding for social mobilization efforts are often not prioritized in constrained district budgets. Advocacy to allocate appropriate resources at the district-level for social mobilization for health services, particularly aimed at the reach hard-to-reach populations, may reap great returns for child health. Continual training and supervision of health workers and community health workers is needed to facilitate community mobilization and to educate mothers on VAS. Healthcare workers should have access to and be familiar with the latest national VAS implementation guidelines.

VAS programs are aimed at reducing child mortality among pre-school aged children. Since Tanzania's VAS Program started in 2001, Tanzania has seen steady declines in under-five child mortality.[9] Unfortunately, this survey indicates that program coverage may be faltering. Post event coverage surveys continue to greatly assist countries in both validating coverage rates as well as identifying weaknesses in programming. The Government of Tanzania's Ministry of Health and Social Welfare can play a critical role in providing national and regional mentorship to ensure tally sheet data accuracy is improved, standardized training curricula are offered to health workers and the importance of the National VAS Program for child mortality is understood by all.

## Acknowledgments

The authors wish to acknowledge the contributions of many who were involved with the implementation of the survey. They include Margaret Benjamin and Temina Mkumbwa from Helen Keller International-Tanzania, Wessy Meghi from Tanzania Food and Nutrition Center (TFNC) and Dr. Fatma Mganga from the Ministry of Health and Social Welfare/Expanded Programme on Immunization (MOHSW/EPI) as well as the time and efforts of the enumerators, health workers, community health workers, village leaders and child caretakers involved in this survey.
